# A three-stage procedure using bone transportation for the treatment of sternoclavicular infectious arthritis

**DOI:** 10.1186/s13018-016-0480-0

**Published:** 2016-11-25

**Authors:** Hua Chen, Xinran Ji, Ming Hao, Qun Zhang, Peifu Tang

**Affiliations:** Department of Orthopaedics Surgery, General Hospital of PLA, Fuxinglu 28, Haidian District, Beijing, 100853 China

**Keywords:** Sternoclavicular infectious arthritis, Three-stage procedure, Bone transportation, Reconstruction

## Abstract

**Background:**

Sternoclavicular joint (SCJ) infectious arthritis is a rare disease. A standard treatment for SCJ infection has not been established. This study aimed to assess the clinical outcomes of a three-stage procedure with bone transportation (BT) for treating SCJ infectious arthritis.

**Methods:**

Six patients (mean age 39.5 years) with chronic SCJ infectious arthritis were included in the study. The patients underwent a three-stage treatment between January 2009 and December 2012, and results were analyzed retrospectively. Following debridement, immediate flap closure was conducted, and BT of the clavicle was performed to fill the gap using a monolateral external fixator. SCJ reconstruction with a tendon autograft was performed, and the external fixator was finally removed. Clinical outcomes were evaluated using Disabilities of the Arm, Shoulder, and Hand (DASH) scores and Constant scores. The average follow-up period was 16 months (range 12–36 months).

**Results:**

The DASH scores decreased from 53.6 ± 4.9 preoperatively to 24.4 ± 3.1 postoperatively. The Constant scores for pain, activity level, positioning, strength, and range of motion were significantly high after the treatment. The total Constant score improved from 32.5 ± 5.8 preoperatively to 76.7 ± 6.4 postoperatively. All patients were satisfied with the therapeutic effect. No complications occurred.

**Conclusions:**

The three-stage procedure with BT improves shoulder function and movement and relieves pain. It is an effective and safe method for treating SCJ infectious arthritis.

## Background

Sternoclavicular joint (SCJ) infectious arthritis is an unusual disease accounting for 1% of all bone and joint infections [1]. Treatment of SCJ infection is difficult because of the close proximity of major vascular structures and lack of substantial overlying soft tissues [2]. Surgery may be performed when conservative treatment fails. Surgical options include incision and drainage, curettage, and/or SCJ resection [2–5]. These procedures require debridement of the structures stabilizing the SCJ, such as the anterior or posterior SCJ ligaments as well as costoclavicular and interclavicular ligaments, with relatively frequent involvement of the first and even second rib [6].

Most patients with SCJ instability complain of discomfort, clicking, and pain because the joint is often dislocated [7–9]. Therefore, an increasing number of surgeons consider it important to restore the joint stability to improve upper extremity function, resulting in the development of other methods such as tendon autograft and resection arthroplasty for SCJ instability [8–11]. A standardized treatment for SCJ infection has not been established. This study proposes a three-stage procedure of debridement, bone transportation (BT), and tendon autografting to restore the clavicular length and SCJ stability. This procedure was utilized in six patients with infectious arthritis. Soft tissue reconstruction was combined with BT following SCJ resection. The study provides novel insights into the management of SCJ infectious arthritis.

## Methods

### Patients

**Fig. 1:**
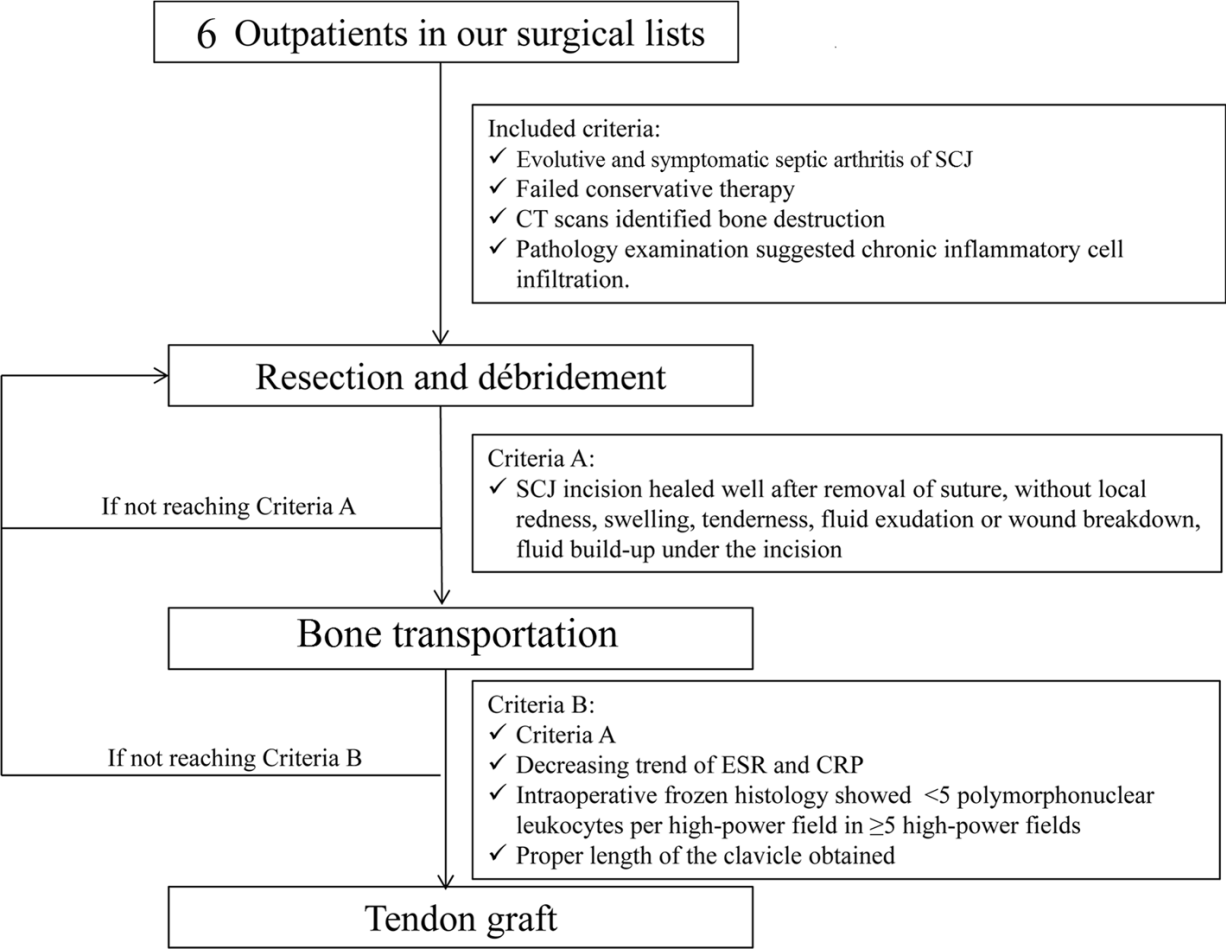
Flowchart of the three stages of the surgical treatment for sternoclavicular joint septic arthritis. *SCJ* sternoclavicular joint, *CT* computed tomography, *ESR* erythrocyte sedimentation rate, *CRP* C-reactive protein

The study enrolled six patients with SCJ infectious arthritis, who met the following inclusion criteria (Fig. 1): evolutive and symptomatic septic arthritis of the SCJ; failure of conservative therapy (immobilization and antibiotic administration); bone destruction detected by computed tomography (CT); and chronic inflammatory cell infiltration determined by puncture biopsy. The patients were treated with a three-stage procedure including BT as described below.

### Operative technique

#### The first stage: resection and debridement

**Fig. 2:**
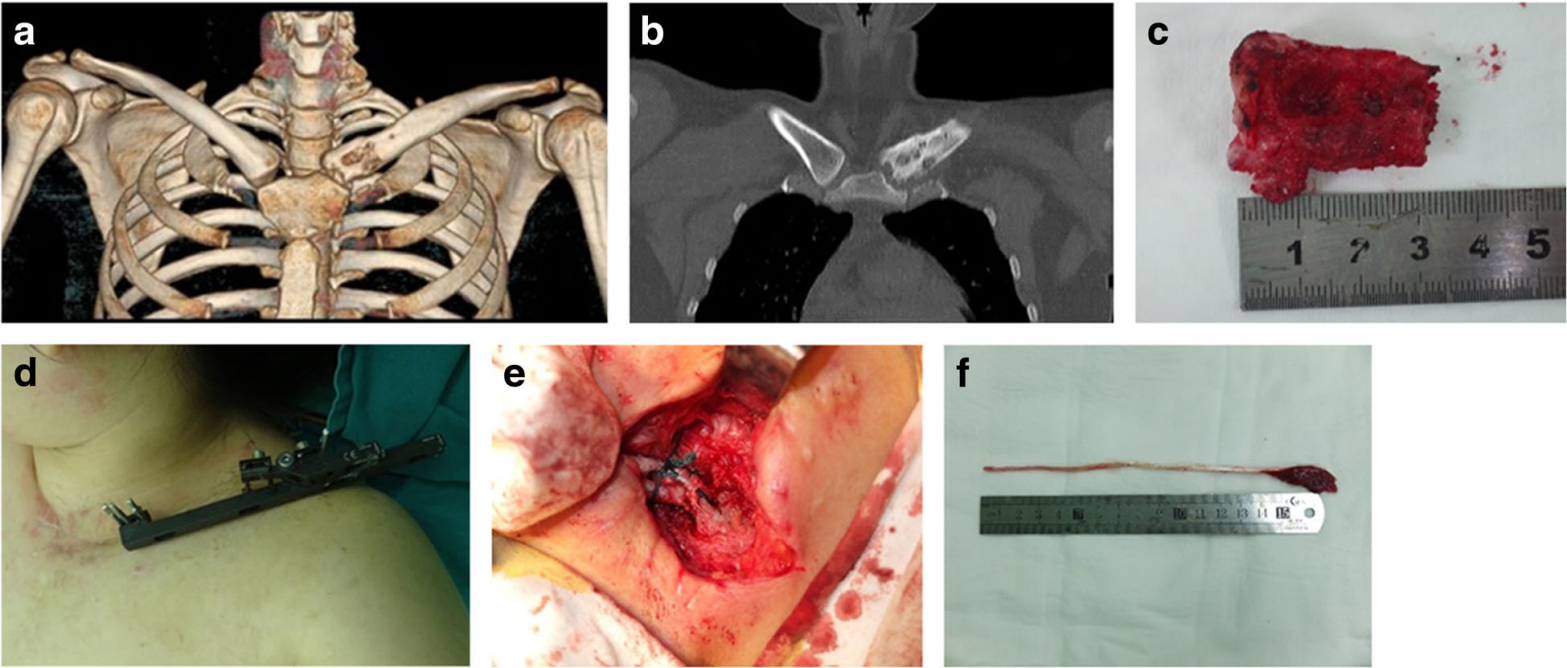
Surgical procedures of the three-stage treatment including debridement, bone transportation, and tendon autografting. **a**–**c** The first stage: resection and debridement. A preoperative CT image shows bone destruction with surrounding inflammation. **d** The second stage: bone transportation. The clavicle length is increased using distraction osteogenesis. **e**, **f** The third stage: tendon grafting. An autologous palmaris tendon graft is placed in a figure-of-eight fashion for restoring the stability of the SCJ

The patient was placed in a beach chair position and was draped to expose the entire length of the clavicle and SCJ. The resection and debridement are shown in Fig. 2a–c. For medial clavicular resection, a 9-cm curved incision centered over the SCJ was made directly. The proximal clavicle, intraarticular disk, and lateral part of the manubrium sterni were removed. The medial end of the first rib was generally debrided with a rongeur. The subclavian vein was protected and pleural entry was avoided if possible. The surrounding infected soft tissue was sharply debrided to healthy margins. A partial pectoralis major advancement flap was next mobilized and inserted to cover the soft tissue defect [2]. One drain was placed under the flap and another superficially to the muscle. The drain was removed when their cumulative output became less than 30 ml/day, and the drainage fluid appeared nonpurulent. The patient was immobilized using a sling and swathe after the surgery, and range of motion and strengthening exercises were started gradually. Antibiotics were used on a case-by-case basis depending on bacterial culture results. Results of the bacterial culture tests were obtained later. After the surgery, antibiotics were administered for 14 days intravenously and then orally for 6 weeks.

#### The second stage: BT

The second stage of the procedure was performed if the patient met criteria A (Fig. 1): the SCJ incision healed well after suture removal, with no local redness, swelling, tenderness, fluid exudation, wound breakdown, and fluid build-up under the incision. If the patient did meet criteria A, debridement was repeated. Stab skin incisions were made to allow for insertion of six 3-mm threaded half-pins into the clavicle, of which three were placed medially and three laterally to the planned osteotomy site. The six bicortical pins were placed parallel to the flat surface of the distal clavicle under fluoroscopy because the longest pin was placed inside the bone with a larger lever arm. An external fixator was attached to the six threaded pins. A 2-cm mid-clavicular oblique skin incision was made at the site of the osteotomy. The periosteum was incised longitudinally, with the cutaneous middle branch of the supraclavicular nerve protected. The subclavius muscle was partially released from the undersurface of the clavicle. A short oblique osteotomy was performed using a 1.5-mm drill and a small osteotome. The wound was then closed in layers with sterile gauze applied to the pin sites. Distraction was commenced on postoperative day 7 at 0.75 mm/day in three separate time intervals. The distraction was continued for 3 to 6 weeks until the length approached that on the contralateral side (Fig. 2d). The fixator was removed when the regeneration was considered stable based on clinical examination and radiology.

#### The third stage: tendon grafting

The third stage of the procedure was performed for patients who met criteria B (Fig. 1): criteria A; decreasing trend in erythrocyte sedimentation rate (ESR) and C-reactive protein (CRP); <5 polymorphonuclear leukocytes per high-power field in ≥5 high-power fields on intraoperative frozen histology; and proper calvicle length. If the patient did not meet criteria B, debridement was repeated. SCJ reconstruction was performed using an autologous palmaris tendon graft after the clavicle length was restored by distraction osteogenesis. Soft tissues between the manubrium sterni and the proximal clavicle were removed. Holes were then made using a drill in the sternum and medial clavicle approximately 15 mm from the joint. With the SCJ in a reduced position, an autologous palmaris tendon was then passed through the drill holes such that it formed a figure 8 (Fig. 2e, f). The repairs were reinforced with nonabsorbable braided polyester suture (Ethibond; Ethicon, Somerville, NJ, USA) applied in a rectangular fashion, which was then tied to it. After the reconstruction, the surrounding periosteum was closed when possible. The patient was immobilized in a sling and swathe for 3 weeks, followed by physical therapy with range of motion and strengthening exercises.

### Clinical evaluation

Functional outcomes were assessed using Disabilities of the Arm, Shoulder, and Hand (DASH) scores and Constant scores preoperatively and at the final follow-up postoperatively [9, 12].

### Bacterial culture and pathological evaluation

The diagnosis of SCJ infection was confirmed by pathological examination using frozen section histology. In the pathological examination, present of at least ten polymorphonuclear leukocytes per high-power field was considered indicative of infection. Presence of fewer than five polymorphonuclear leukocytes per high-power field reliably indicated absence of infection. The bacterial cultures for oxacillin-sensitive *Staphylococcus aureus* were performed to determine whether the antibiotic therapy had been successful. Cultures for oxacillin-sensitive *S. aureus* were performed to obtain definitive results even if a type I incision surgery was conducted during the second stage (BT). Pathological examinations and bacterial cultures were performed before admission and at the three stages of the surgery.

### Radiological evaluation and follow-up

Radiographic examinations were performed to monitor progress every 2 weeks after the BT surgery until the clavicle attained its proper length to monitor its progress. The external fixator was adjusted when unsatisfactory alignment was detected. Serial anteroposterior radiographs were obtained 3 weeks after the BT and then every 4 weeks throughout the distraction and consolidation phases to monitor the regeneration. Radiographic examinations were performed every 4 weeks after the tendon graft surgery to monitor the consolidation of the regenerated area. CT and three-dimensional reconstruction were carried out after removing the external fixator. The follow-up period started after the last stage of the surgery.

### Statistical analysis

Values are expressed as mean ± SD (standard deviation). Student’s *t* test was used for comparison of groups before and after surgery, if necessary. A *p* < 0.05 was considered to indicate a significant difference.

## Results

### Basic characteristics of the included patients

The details of the three-stage procedure with BT are presented in the flow diagram (Fig. 1). The demographic and clinical data of the six patients are shown in Table 1. There were four men and two women, with a mean age of 39.5 years. The left SCJ was affected in four patients, and the right SCJ was affected in the remaining two patients. The mean follow-up period was 16 months (12–36 months).

**Table 1:** Basic information for patients included in this study

Case number	Sex	Age	Affected joint	Bone loss from the initial debridement (mm)	Time of lengthening the bone (days)	Overall bone length gained (%)	Duration of external fixator (days)	External fixator index (days/cm)	Time between 1st and 2nd procedures (days)	Time between 2nd and 3rd procedures (days)
1	F	37	L	32	41	21.3	160	50	21	46
2	F	38	R	35	50	27.3	180	51	27	53
3	M	38	L	41	56	30.1	220	52	30	60
4	F	45	R	30	46	22.9	170	55	36	50
5	M	42	L	33	45	26.2	187	57	27	52
6	F	37	L	36	44	24.6	178	49	29	48

*F* female, *M* male, *L* left, *R* right

### Clinical results

After the surgery, all the patients returned to full activity without limitations. The clinical results of each patient are shown in Table 2. The DASH scores decreased from 53.6 ± 4.9 preoperatively to 24.4 ± 3.1 postoperatively (*p* < 0.05). Analysis of Constant score revealed that the scores for pain, activity level, positioning, strength, and range of motion (ROM) at the last follow-up were significantly higher than the corresponding preoperative scores. Total Constant score improved from 32.5 ± 5.8 preoperatively to 76.7 ± 6.4 at the last follow-up (*p* < 0.05).

**Table 2:** Clinical results in this study

Variable	Case number												Mean (SD)	
	1		2		3		4		5		6			
	Preop	Last follow-up	Preop	Last follow-up	Preop	Last follow-up	Preop	Last follow-up	Preop	Last follow-up	Preop	Last follow-up	Preop	Last follow-up
Constant score														
Pain (0–15)	5	15	5	15	5	15	0	10	0	15	0	10	2.5 (2.7)	13.3 (2.6)*
Activity level (0–10)	0	10	0	10	2	10	0	8	2	10	2	8	1.0 (1.1)	9.3 (1.0)*
Positioning	6	8	6	8	6	8	6	8	8	8	6	10	6.3 (0.8)	8.3 (0.8)*
Strength (0–25)	2	14	5	17	5	14	5	17	11	23	2	17	5.0 (3.3)	17.0 (3.3)*
ROM (0–40)	18	30	20	30	16	28	14	24	20	30	18	30	17.7 (2.3)	28.7 (2.4)*
Total score (point)	31	80	36	80	34	75	25	67	41	86	28	75	32.5 (5.8)	77.2 (6.4)*
DASH (100–0)	51.6	23.3	55	23.3	52.5	23.3	60.1	30.8	45.8	23.3	56.7	22.5	53.6 (4.9)	24.4 (3.1)*

Values in parentheses are the SD (standard deviation). Student’s *t* test was used for all statistical analyses

*ROM* range of movement, *Preop* pre-operation

*Statistically significant difference between preoperative and last follow-up

Neither the gross strength nor the ROM was limited at the final follow-up. All the patients were satisfied with the therapeutic effects.

### Pathological and bacterial culture results

The bacterial culture tests identified oxacillin-sensitive *S. aureus* as the source of the SCJ infection. Three patients who were positive for oxacillin-sensitive *S. aureus* before admission were found to be negative starting from the stage of resection and debridement. Pathological examination revealed that all the patients had infection before admission and during the resection and debridement, which might have been caused by oxacillin-sensitive *S. aureus.* No infection was present in any patient during the BT surgery and tendon graft surgery.

### Radiographic results

**Fig. 3:**
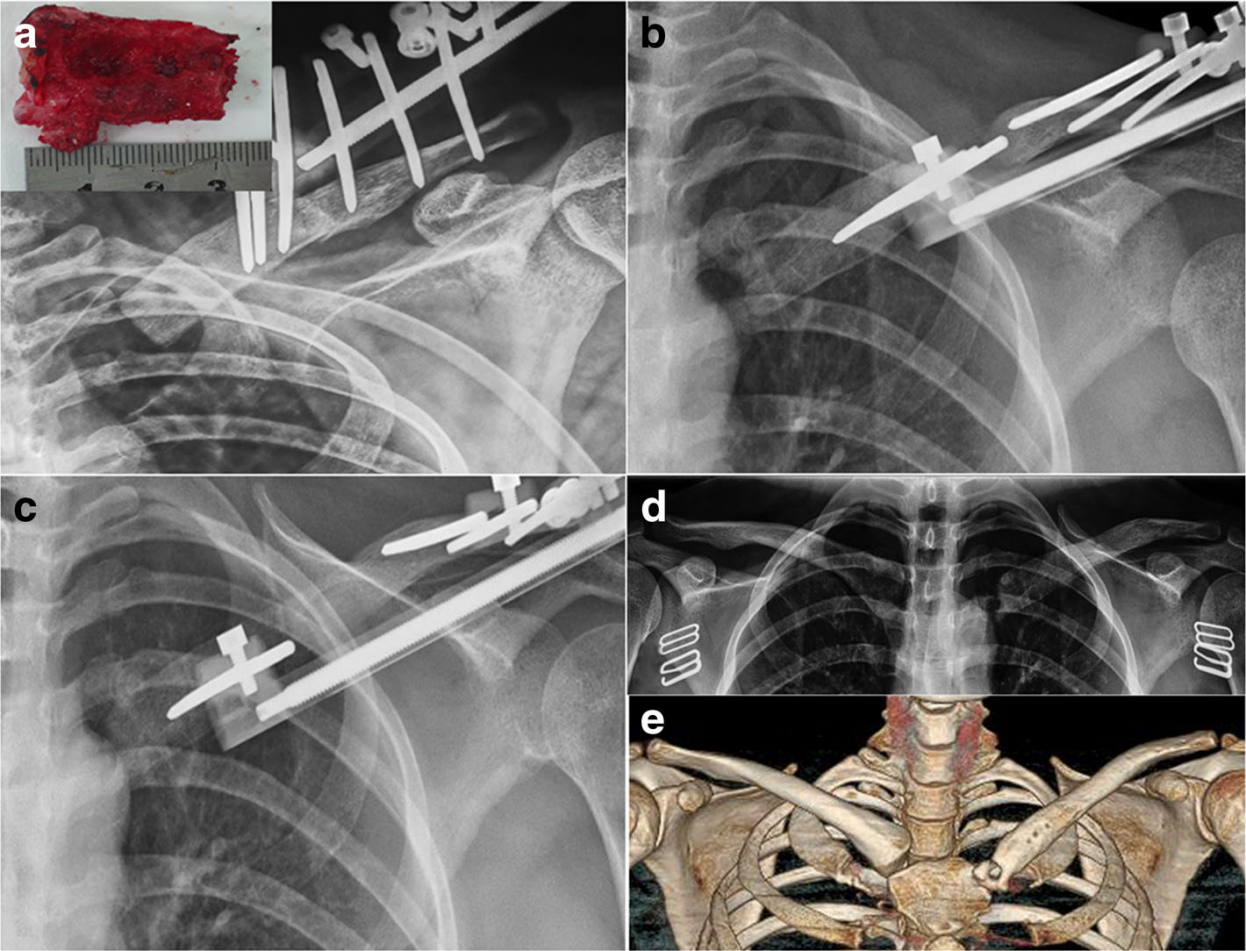
Radiographic results. **a** A radiograph with a 20° cephalic tilt of the right clavicle taken immediately after the surgery shows the osteotomy site and the 35-mm resection of the proximal clavicle during debridement of the SCJ. **b** An anteroposterior (AP) radiograph of the right clavicle taken at postoperative week 10 shows regeneration after distraction at 0.75 mm/day. **c** An AP radiograph of the right clavicle taken at week 26 shows consolidation of the regenerated area. **d**, **e** An AP radiograph and computed tomography scan with three-dimensional reconstruction of the right clavicle taken at week 56 after the removal of the external fixator demonstrates the sustained restoration of the clavicle length and the integrity of the sternoclavicular joint

Postoperative radiographic examination showed satisfactory alignment in all cases. The bone healed in all the patients after removal of the external fixator. There was no need for internal fixation or bone grafting (Fig. 3).

### Complications

No problems or complications, such as pain or bleeding were reported postoperatively. The SCJ infections did not recur in any patient. There was no need for revision in any patient.

## Discussion

In the current study, a three-stage procedure with BT was used to treat six patients with SCJ infectious arthritis. The infection was controlled and the wound healed after the SCJ debridement. Following the BT, the clavicle length was restored using distraction osteogenesis. The six patients then underwent tendon autograft reconstruction of the SCJ without internal fixation. The mean follow-up was 16 months. It was found that only three patients were positive for oxacillin-sensitive *S. aureus* preoperatively. This indicates that the SCJ infection might be caused by other organisms. More experiments would be needed to elucidate the etiology. The DASH scores decreased and the Constant scores improved remarkably after the surgery. All the patients were satisfied with the therapeutic effect. No complications occurred postoperatively. These results suggest that the three-stage procedure with BT is effective and safe for treating patients with SCJ infectious arthritis.

An increasing number of studies have recommended several techniques for SCJ reconstruction, such as the figure-of-eight technique [13], tendon of the sternocleidomastoid muscle grafting [14], and sternocleidomastoid tendon grafting [15]. For patients with SCJ infection, SCJ debridement should be conducted first. SCJ reconstruction can then be after the infection is controlled and the wound has healed.

There is currently on consensus on the best option for SCJ infectious arthritis. Song et al. found that simple incision, drainage, and debridement were ineffective, with possible recurrence of the infection [2]. Another method capable of eradicating bone infection has been proposed for treating long bone infections, which includes wide debridement and placement of an antibiotic-loaded cement spacer followed by cancellous bone grafting during a second-stage operation [16–18]. However, this method is unsuitable for SCJ infection because the extent of movement in the joint would hamper bone union between the cancellous graft placed in the gap and the proximal clavicle. Another problem is the difficulty of obtaining stable fixation between the cancellous graft and the proximal clavicle. These problems have been solved successfully in the present study. Mechanical stretching employed in the BT approach stimulates new bone formation in the gap between the gradually distracted fragments, and the gap is filled. BT is a safe technique for gradually restoring the bone to its original length. It uses the principles of distraction osteogenesis, as described by Ilizarov [19]. To our knowledge, the present study presents the first successful implementation of the BT technique for treating SCJ infection. Bone healing was achieved in all the patients after the removal of the external fixator without the need for internal fixation or bone grafting. Moreover, neither the gross strength nor the ROM was limited on the affected side. All the patients returned to full activity without limitations.

The three-stage procedure with BT combined debridement, BT, and tendon autografting. Tendon autografting was the third treatment stage in the present study. Bak et al. have reported that mini-open SCJ reconstruction using a tendon autograft can markedly improve the shoulder function of most patients with symptomatic anterior SCJ instability, although 68% of patients complained of donor-site morbidity, and 40% still had some discomfort at follow-up [8]. In contrast, the DASH scores decreased and Constant scores improved remarkably after the three-stage procedure with BT. Furthermore, a satisfactory therapeutic effect was achieved in every patient. These findings indicate that the three-stage treatment with BT may be more effective and safer than SCJ reconstruction using a tendon autograft in patients with SCJ infectious arthritis.

In the study of Sewell and colleagues, the mean gradual clavicular length gained with distraction was 31 mm (15–41 mm), which corresponded to an average of 24.7% of the overall bone length. According to the literature, distraction exceeding 25% of the overall bone length may require additional plate fixation to consolidate the union [20]. In our study, the resected clavicular length (mean 35 mm) was greater than that in the report of Sewel et al. However, the osteotomy site was not augmented with a plate and a bone graft, avoiding the risk and disadvantages with using an internal fixator.

A previous study has reported that three patients who underwent distraction at a rate of 1 mm/day required augmentation with a clavicular plate after fixator removal to prevent deformation and fracture of the regenerated bone [20]. A relatively slow distraction might allow the regeneration of more blood vessels and periosteal cells in the gap created, thereby preventing the surrounding soft tissue from entering the gap. In the present study, the rate of distraction was 0.75 mm/day, and we did not encounter any problems that would require augmentation with a plate or a bone graft.

Although no complications occurred in the six patients in this study, the three-stage procedure of debridement, BT, and tendon autografting may cause some adverse effects, such as thoracic aorta injury and nerve injury. The important organs surrounding the SCJ are at risk of injury as well. Therefore, much attention should be paid to avoid complications when conducting the three-stage surgery.

The present study has several strengths. Firstly, it presents a novel approach wherein a combination of soft tissue reconstruction and BT is used after resection of the SCJ. Secondly, it suggests that distraction osteogenesis can be used not only in long bone defects but also in short and thin bone defects, in particular, in the case of the clavicle. The study also has some limitations. This method requires that the patients wear an external fixator for about half a year, which can cause inconveniences. Moreover, the number of patients was small, and a control group without lengthening was not employed. DASH and Constant scores were not measured prior to the second procedure (BT) to determine whether improvements were due to the three-stage procedure or simply the excision. Additionally, some recall bias might exist in both the clinical and functional data that we collected. Further studies will address these issues and validate the findings of this study.

## Conclusions

The three-stage procedure for treating SCJ infectious arthritis efficiently improves shoulder function, relieves pain, and improves movement. It may serve as a new surgical treatment for patients with SCJ infectious arthritis.

## Abbreviations

BT: Bone transportation
CRP: C-reactive protein
CT: Computed tomography
DASH: Disabilities of the Arm, Shoulder, and Hand
ESR: Erythrocyte sedimentation rate
ROM: Range of motion
SCJ: Sternoclavicular joint
